# Resistance to Moisture-Induced Damage of Half-Warm-Mix Asphalt Concrete with Foamed Bitumen

**DOI:** 10.3390/ma13030654

**Published:** 2020-02-01

**Authors:** Mateusz M. Iwański, Anna Chomicz-Kowalska, Krzysztof Maciejewski

**Affiliations:** 1 Department of Building Engineering Technologies and Organization, Faculty of Civil Engineering and Architecture, Kielce University of Technology, Al. Tysiąclecia Państwa Polskiego 7, 25-314 Kielce, Poland; 2 Department of Transportation Engineering, Faculty of Civil Engineering and Architecture, Kielce University of Technology, Al. Tysiąclecia Państwa Polskiego 7, 25-314 Kielce, Poland; akowalska@tu.kielce.pl (A.C.-K.); kmaciejewski@tu.kielce.pl (K.M.)

**Keywords:** foamed bitumen, half-warm-mix asphalt, moisture and frost resistance, hydrated lime

## Abstract

Hot-mix asphalt (HMA) remains the predominant material for pavement surfacing. Mixing is performed at about 180 °C, depending on the bitumen used. Environmental concerns in terms of emissions and energy demand are fostering new sustainable technologies in road construction. Warm-mix asphalt (WMA) and half-warm-mix asphalt (HWMA) mixtures meet current expectations in that they are produced at lower temperatures, 100–130 °C, ensured by foaming the bitumen with water. The extent of temperature reduction requires that the mixture has adequate moisture and frost resistance, which is particularly important in countries that have a low-temperature climate. Asphalt concrete AC 8 S with 50/70-grade foamed bitumen modified with 0.6 wt.% surface-active agent (SAA) was used in the tests. To provide the AC mixture with the required resistance to climatic factors (water, temperature below 0), hydrated lime was added at 0, 15, 30, and 45 wt.% as limestone filler replacement. The influence of the hydrated lime addition on the air void content and resistance to moisture and frost damage was investigated according to the WT-2 2014 methodology based on EN 12697-12: 2008 and to the modified AASHTO T283 method. The optimum content of hydrated lime for filler replacement was determined through statistical analysis of the test results. With the optimum hydrated lime replacement of 30%, the required level of moisture and frost resistance of HWMA concrete with foamed bitumen is achieved. The results of this study confirmed the suitability of HWMA concrete with foamed bitumen for application in road construction practice.

## 1. Introduction

The sustainability of road construction processes is understood as minimizing negative environmental impacts through reducing greenhouse emissions and energy use of asphalt mixing plants. Conventional hot-mix asphalt (HMA) mixtures are produced at high temperatures reaching 180 °C, depending on the bitumen type used. During production, a large amount of CO_2_ and other harmful gases are emitted, and a large amount of energy is needed to dry the aggregate and bring the bitumen from solid to liquid with a viscosity of about 0.2 Pa∙s. The need to find more eco-friendly technologies has been reflected in the number of research and development activities leading to the implementation of low-temperature processes. Warm-mix asphalt (WMA) is produced and placed at lower temperatures compared with HMA [1,2]. This temperature reduction results from adding a variety of chemical agents [3,4,5,6,7], such as low-viscosity additives that decrease bitumen viscosity [8,9,10,11,12] and foamed bitumen based on natural [13,14,15] or synthetic zeolite [16,17].

In half-warm-mix asphalt (HWMA) technology, the lowering of temperature is even more pronounced. The binder is the bitumen foamed with water which lowers the binder viscosity [18,19,20,21,22,23] and the process temperature is lower by about 40–60 °C in comparison with traditional HMA. A HWMA mixture with foamed asphalt and a traditional mixture should have comparable properties [24,25,26]. Until recently, foamed bitumen was used only in deep cold recycling [20,27,28] and in this respect, quality requirements were set for the foaming parameters: maximum expansion—ER, and half-life—HL of bitumen foam [20,29]. 

Ensuring high parameters of foamed bitumen is critical for achieving the required parameters of the mixture. Research is being carried out on the modification of the binder before foaming using synthetic Fischer–Tropsch wax [30,31] or surfactants [32]. The use of wax causes certain technological difficulties at a temperature below 100 °C. At this temperature, the F–T wax crystallizes in the binder causing compatibility problems [11,25]. These problems can be avoided by adding a surface-active agent (SAA) as a binder additive [32]. The field practice shows, however, that the use of SAA in HMA mixtures not only fails to enhance the standard properties but may have a slightly adverse effect in some cases [33]. Based on HMA practice, the use of hydrated lime as a partial filler replacement in HWMA mixtures could present beneficial effects. 

The idea of using lime in bituminous mixtures is not new. The forerunner in this field was the United States [34,35] more than a hundred years ago. Research carried out over several subsequent decades has managed to prove the positive role of hydrated lime in traditional HMA mixtures [36,37]. Lime was shown to increase moisture and frost resistance [38,39] as a result of improving the bitumen-aggregate adhesion [38,39,40].

Hydrated lime plays an important role in slowing the ageing of bitumen and bituminous mixtures, thereby extending the durability of pavements for their moisture and frost resistance and the resistance to permanent deformations [41,42,43].

The addition of hydrated lime reduces age-hardening of bitumen, which, as a result, have lower stiffness [40]. The type of bitumen has a direct influence on the intensity with which lime reduces age-hardening of the binder [39,44,45,46].

Research on the binder recovered from bituminous mixtures used in structural layers is important for identifying the effect of hydrated lime on bitumen properties [40,47,48]. The recovered binder was found to have higher viscosity than lime-free binders [40].

Hydrated lime significantly reduces the ageing process of bituminous mixtures regardless of their composition [49], which is important in the process of ensuring freeze-thaw resistance of asphalt concrete [50,51].

Development of WMA technology requires that various types of additives and modifiers are used to obtain the required mixture quality, primarily in terms of its resistance to permanent deformations and frost resistance performance, which may be decreased due to lowered mix processing temperatures [26]. Hydrated lime has also been used for this purpose. However, experience in this area is very limited as only a few studies have shown positive effects of hydrated lime in the mixture composition on ensuring its resistance to moisture and frost [52,53]. 

As it is a multifunctional additive, hydrated lime ensures durability of bituminous surfaces by improving moisture and frost resistance of bituminous mixtures and structural pavement layers and by increasing pavement resistance to permanent deformations [40]. It can also contribute to achieving the required level of the material characteristics of HWMA mixtures with foamed bitumen. 

The use of foamed binders with the highest parameters in HWMA mixtures for extending the road work season makes it necessary to add bitumen modifiers (SAA) before foaming. This technology, in addition to ensuring high bitumen foaming characteristics, should also guarantee that the mixture meets the required level of moisture resistance. To achieve this, hydrated lime can be used as in traditional HMA mixtures. The aim of this study was to assess the influence of foamed bitumen binder and hydrated lime fraction in the mineral filler on providing moisture and frost resistance of asphalt concrete intended for the wearing course, produced at a reduced temperature in the HWMA process.

The moisture and frost resistance of the mixture is one of the main factors that influence the durability of the asphalt pavement [54]. This factor should be carefully considered when new material solutions are introduced, and limited laboratory or field experience is available. A detailed analysis of the applied research methods is necessary to allow proper assessment of the resistance of the mixture to the climatic factors. Research experience shows that depending on the procedures adopted, the methods can be assessed in a variety of ways [55,56]. Therefore, the moisture and frost resistance of the produced mixtures was evaluated using two different procedures, using one and 18 freeze-thaw cycles, respectively. Another objective was to optimize HWMA concrete composition.

## 2. Tested Materials

### 2.1. Experimental Program

The tests aimed at determining the influence of hydrated lime on the durability of HWMA mixtures with foamed bitumen. Two moisture damage testing procedures that significantly differ in conditioning technique were used. The procedure recommended by the Technical Requirements WT-2 is less severe than the AASHTO T283 modified method. Analysis of the AC 8 manufacturing technology effects included determining the parameters below as per WT-2 2014 [57] and EN 13108-1:2008:

✓ air void content (*V_a_*, %) to EN 12697-8:2005,
✓ water sensitivity (*ITSR*, %) to WT-2 2014 [58],
✓ resistance to moisture-induced damage (*RW_WM_*, %) to the modified AASHTO T283 method [51].

Parameters *V_a_*, *ITSR* and *RW_WM_* were determined by compacting the specimens using a Marshall hammer with the number of blows depending on the procedure used. All characteristics were determined on a total of nine specimens [59] that met the required physical and geometrical criteria [58].

#### 2.1.1. Air Void Content (V_a_)

The air void content is the volume of air voids in the bituminous specimen, expressed as a percent of the total volume of that specimen. This parameter was determined as per EN 12697-8:2005 using the formula below

(1) $$V_{a}=\frac{\rho_{m}-\rho_{b}}{\rho_{m}}\times 100\%$$

where:

Va>V_a_—volume of air voids in the bituminous specimen (vol %),
*ρ_m_*—density of the bituminous mixture (kg/m^3^),
*ρ*_b_—bulk density of the bituminous mixture (kg/m^3^).

#### 2.1.2. Water Sensitivity (ITSR) in Accordance with WT-2 [58]

The test was performed on Marshall specimens 63.5 mm ± 2.5 mm high and 101 mm in diameter. The compaction procedure consisted of applying 35 blows to each face of the specimen in the Marshall apparatus.

The prepared specimens were divided into two equal sets before conditioning. One set was stored at room temperature (20 ± 5 °C) without additional conditioning (so-called "dry set"). The other set (the so-called "wet set") was conditioned according to the WT-2 2014 procedure. 

Conditioning of the specimens from the "wet set" began with placing them on a perforated shelf in a vacuum tank (chamber) filled with distilled water at a temperature of (20 ± 5 °C). After immersion, their upper surfaces were at least 20 mm below the water level. Then the vacuum apparatus was activated and absolute pressure (6.7 ± 0.3) kPa was obtained within 10 ± 1 min. To avoid damage to the specimens, the pressure was lowered slowly and evenly, and then maintained for a period of 30 ± 5 min. After that, the pressure was slowly increased up to the level of atmospheric pressure and then the specimens were left in water for another 30 ± 5 min. After removing from water, the dimensions of the specimens were measured in accordance with EN 12697-29: 2006 and their volumes were calculated. Specimens that increased their volume by more than 2% were discarded and the degree of water saturation was determined according to the formula:

(2) $$N_{w}=100\times \left(B-A\right)/\left[V-\left(B-C\right)\right].$$

where:

*N_w_*—degree of water saturation of the specimen [%],
*A*—air-dry specimen mass before vacuum saturation [g],
*B*—specimen mass in air after vacuum saturation, [g] after surface drying,
*C*—specimen mass in water after vacuum saturation [g],
*V*—air void content in the sample, expressed as a decimal number.

The specimens with a saturation level above 80% were discarded. If the saturation level was below 55%, the saturation procedure was repeated. Specimens from the "wet set" were placed in a 40 ± 1 °C water bath for a period of 68 h. After removal from the water bath, the dripping wet specimens were tightly wrapped in a stretch film and placed in a plastic bag containing (10 ± 1) mL of water, then sealed and frozen at −18 ± 3 °C for a minimum of 16 h, counting from the time the freezer reached the required temperature. From the freezer the specimens were moved to a 25 ± 2 °C water bath. Shortly after submerging the specimens in water, they were removed from the plastic bags, unwrapped and quickly placed back in the water for 24 ± 1 h from the time of first insertion into the water after storage in the freezer. After conditioning, the indirect tensile strength of all the specimens was determined according to EN 12697-23.

The moisture and frost resistance indicator, ITSR, was calculated using the formula:

(3) $$ITSR=\frac{ITS_{d}}{ITS_{w}}\times 100\%$$

where:

*ITS_d_*—average indirect tensile strength of air-conditioned specimens,
*ITS_w_*—average indirect tensile strength of water-conditioned specimens as per WT-2 2014.

#### 2.1.3. Resistance to Moisture-Induced Damage in Accordance with Modified AASHTO T283 Method

The tests were conducted in accordance with the modified AASHTO T283 method [47,48,50,51] on Marshall-compacted specimens 63.5 mm ± 2.5 mm high and 101 mm in diameter. The compaction procedure was modified to obtain 6% to 8% voids. The prepared two sets of specimens differed by conditioning method. The dry set was air-conditioned at room temperature until testing. 

The wet set specimens were placed in a vacuum chamber filled with distilled water. The upper surface of the specimens was covered by at least 20 mm of water. The pressure in the vacuum chamber was reduced to reach 6.7 kPa for 30 min. Then the specimens were put to a 40 °C ± 1 °C water bath for 68 h. After that, the specimens were wrapped in a plastic foil and frozen at −18 °C for at least 4 h, then thawed for 4 h at 20 °C. The number of freeze-thaw cycles was 18 as recommended by the adopted procedure [50,51]. After removal from the freezer, the specimens were submerged in water at 60 °C ± 1 °C for 24 h.

Directly before conditioning, all the specimens were stored in a 25 °C water bath for 3 h. The indirect tensile strength test at 25 °C was run in the universal testing machine at a rate of 50 mm/min. The load was applied to failure by caps 12 mm in width and with 50.5 mm curvature radius. The loading scheme was identical to that used during the indirect tensile strength test. The ratio for moisture-induced damage RW_WM_ according to the modified AASHTO T283 procedure was calculated from

(4) $$RW_{WM}=\frac{ITS_{w}^{A}}{ITS_{d}^{A}}$$

where:

*ITS_d_^A^*—average indirect tensile strength of air-conditioned specimens,
*ITS_w_^A^*—average indirect tensile strength of water-conditioned specimens undergoing a freeze-thaw cycle as per AASHTO T283,

### 2.2. Materials and Mix Design Procedure

In the laboratory tests, 5.6%, 5.9%, 6.2% and 6.5% foamed bitumen was used in the HWMA concrete mixture, as required for the wearing course. [58] The bitumen was a 50/70 paving-grade bitumen, commonly used in the countries of central and eastern Europe in bituminous mixes for road pavements under traffic characterized by 2.5 × 10^6^ < ESAL_100 kN_ < 7.3 × 10^6^ (ESAL—equivalent single axle load) [58].

For the use in HWMA, the 50/70 bitumen was modified with fatty acid amide-based SAA in the amount of 0.6 wt.% (in relation to the mass) of the binder. The SAA properties are summarized in Table 1.

The bitumen containing 0.6% SAA was subjected to water-based foaming. The foam characteristics, expansion ratio ER [19,29] and half-life HL [19,29], were determined with 9 replicates in the second stage of the study [59].

Physical properties of the foam were tested in a Wirtgen WLB-10S foaming plant by applying different foaming water contents (FWC): 1.5%, 2.0%, 2.5%, 3.0% and 3.5% by weight as per [29].

The test results of unmodified and 0.6% SAA modified bitumen are compiled in Table 2.

**Figure 1:**
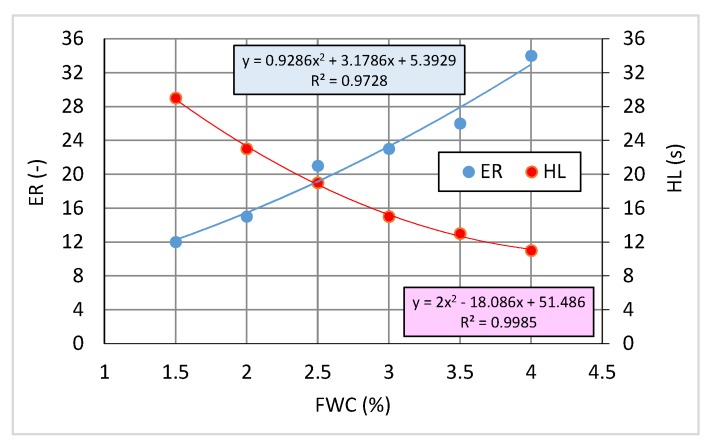
Characteristics of 0.6 % SAA modified bitumen 50/70.

Figure 1 shows the characteristics of 50/70-grade foamed bitumen after 0.6% SAA modification. 

The 50/70-grade bitumen containing 0.6% SAA has very high foaming parameters. Hence, its use in the mineral asphalt concrete mixture should ensure that all aggregate particles are well coated. Detailed results of the study of SAA modified bitumen 50/70 were described in [32].

**Figure 2:**
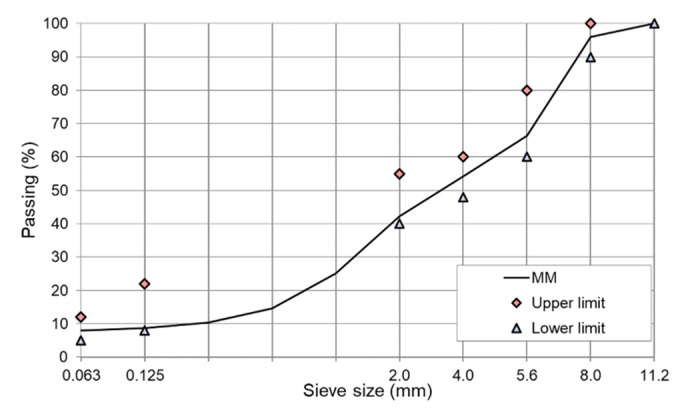
Grading curve of AC 8 mineral mixture with limiting points as in WT-2 2014 requirements [58].

The basic frame-compositions of the mineral mixture and bituminous mixture are summarized in Table 3 and the particle size design of AC 8 S is plotted in Figure 2.

The research used a variable bitumen contents to determine the effect of the bitumen amount on asphalt concrete properties in terms of hydrated lime dosing. While increasing the amount of foamed bitumen from 5.9% to 6.5% according to the experimental design, the grading of the mineral mixture was adjusted accordingly. 

To ensure moisture resistance of the asphalt concrete, hydrated lime was added at 15%, 30% and 45% by weight as a lime filler replacement. 

The AC 8 S mixture was prepared in a heated 60 l mechanical mixer to which foamed bitumen produced in the WLB-10S plant was added. The composite was mechanically mixed with a stirrer for a maximum of 5 min. The production temperature of AC 8 S with additives did not exceed 100 °C.

The mixture prepared in this way was compacted using the Marshall impact compactor. The number of impacts was dependent on the test type. After making, the asphalt concrete specimens were allowed to cool at room temperature for 48 h.

**Figure 3:**
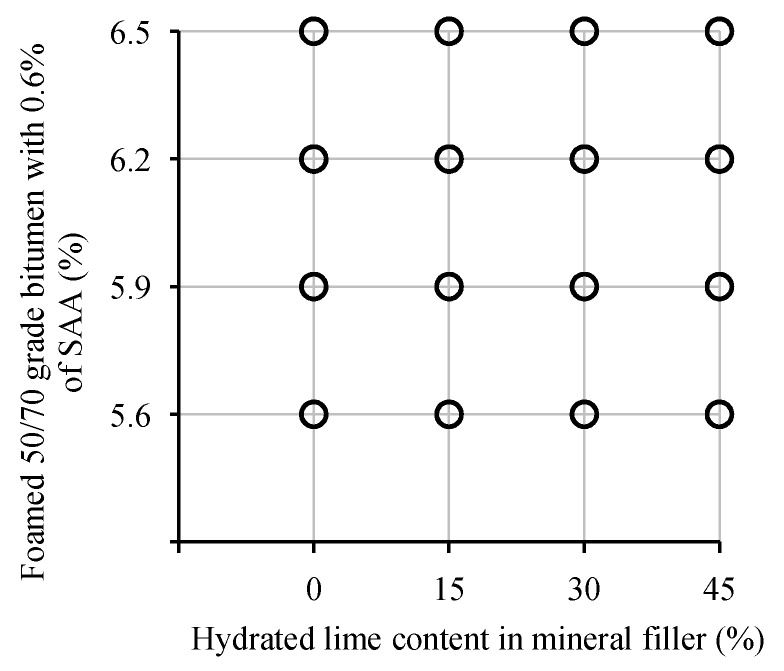
Experimental design.

The algorithm for the design of factorial experiments was used [60]. The physical and mechanical parameters of AC 8 S were determined based on the adopted 4 × 4 factorial design, in accordance with the testing program. The developed plan of the experiment is shown in Figure 3. 

## 3. Results and Discussion

### 3.1. The Effects of the Foamed Bitumen and Hydrated Lime on Air Void Content in Asphalt Concrete

Air voids play an important role in shaping the structure of the bituminous mixture. The amount of air voids has a large impact on the material properties of asphalt concrete. Too high an air void content negatively affects moisture resistance and performance under high loads. On the other hand, the insufficient air void content decreases the resistance of the mixture to permanent deformations despite being beneficial in terms of moisture resistance. Therefore, when examining new bituminous materials or their modifications, particular attention should be paid to this parameter. HWMA mixtures should have an air void content comparable to that obtained by traditional hot-mix asphalts if proper operation in asphalt pavement and durability are to be ensured.

The air void content of the mixture was studied in accordance with the presented methodology as per EN 12697-6: 2008. Asphalt concrete AC 8 S should have 2.0% to 4.0% air voids [58]. Test results of the AC 8 HWMA mixture with 0.6% SAA modified foamed bitumen and hydrated lime are summarized in Table 4 together with the basic statistical parameters. 

**Figure 4:**
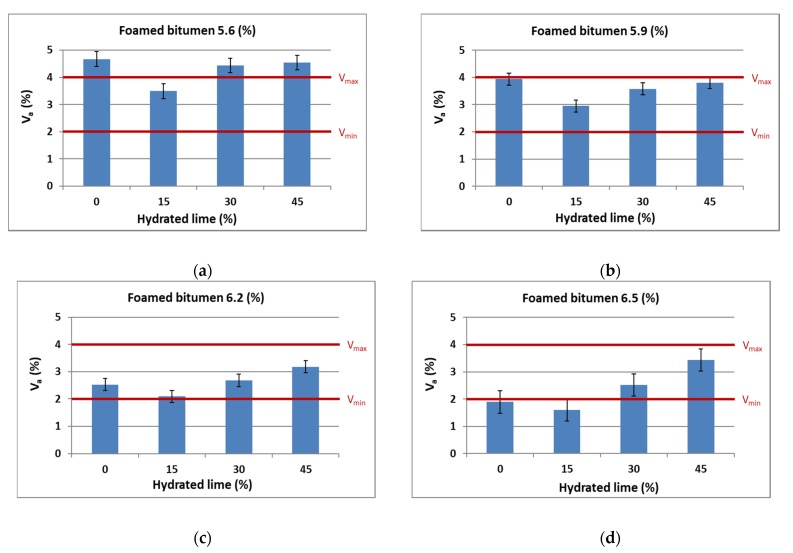
Air void contents versus the quantity of hydrated lime and foamed bitumen at: (**a**) 5.6%, (**b**) 5.9%, (**c**) 6.2%, (**d**) 6.5%.

Graphic representation of the quantity of hydrated lime and foamed bitumen on the quantity of air voids V_a_ in AC 8 S is shown in Figure 4.

As the amount of foamed bitumen in AC 8 S mixtures increases to 6.2%, the air void content in asphalt concrete decreases, which is consistent with the general trend in this respect. However, with the binder amount of 6.5%, the air void content increases, which results from excessive binder content in the mix and its segregation. The use of 15%, 30% and 45% hydrated lime to replace mineral lime filler significantly influences variations in the value of this parameter. The addition of 15% m/m hydrated lime reduces the content of air voids, which is certainly the effect of improved adhesion of the mineral mixture-bitumen interface. Increasing hydrated lime content to 45% m/m leads to an increase in the air void content. This can be attributed to insufficient amount of binder due to the use of hydrated lime, which has a larger specific surface area than the lime filler. As a result, compaction of the mixture becomes difficult and the content of air voids increases [61]. Regardless of the hydrated lime content, AC 8 S containing 6.2% foamed bitumen has the smallest amount of air voids. This binder content may impede maintaining adequate resistance to permanent deformations [24,25].

To comprehensively describe the variations in the AC 8 S air void content due to the changed content of the 50/70 foamed bitumen with 0.6% SAA and hydrated lime content, a statistical model using a second-degree polynomial [62] was adopted:

V_a_ = b_0_ + b_1_x_1_ + b_2_x_2_ + b_3_x_1_x_2_ + b_4_x_1_^2^ + b_5_x_2_^2^ (5)

where: x_1_ = foamed bitumen − FB (%), x_2_ = hydrated lime − HL (%), b_0_–b_5_: regression coefficients 

In the first step, a significance test was used with analysis of variance (ANOVA) [63] (Table 5).

Analysis of the parameters listed in Table 5 indicates clearly that the content of foamed bitumen and hydrated lime is an important factor that has an effect on the air void content in the AC 8 S mixture, as demonstrated by the *p*-value being lower than the pre-set significance level α = 0.05 (values in red). An interaction effect is present between the content of foamed bitumen and hydrated lime, which affect the air void content in the mixture (*p*-value less than α = 0.05).

The values describing the parameters of the regression model for the relationship between the air void content in terms of the amount of foamed bitumen and hydrated lime are summarized in Table 6. 

Analysis of the procedure used shows that the value of the adjusted coefficient of determination R^2^ is 88% which confirms the adequacy of the model. The amounts of foamed bitumen and hydrated lime as well as the interaction of these factors have a significant impact on the air void content in the mixture.

**Figure 5:**
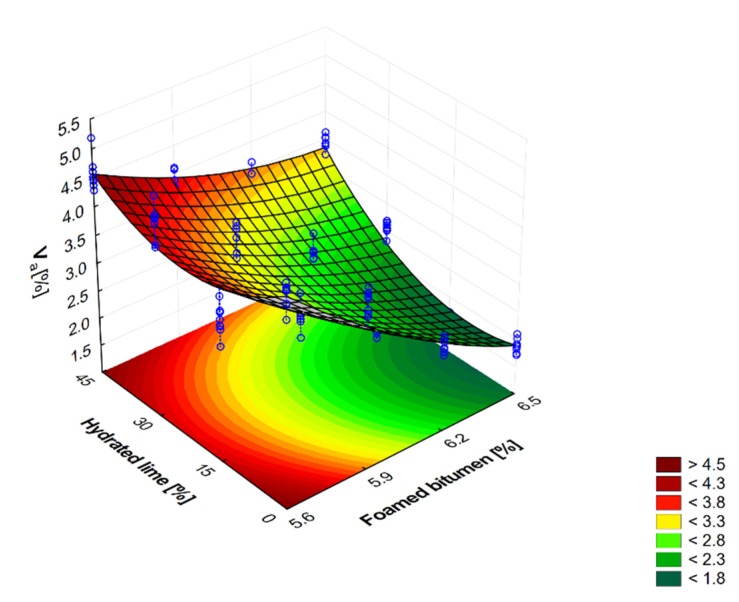
Air void content V_a_ in AC 8 S mixture versus the contents of foamed bitumen and hydrated lime, and the representation of the model describing the relationship. V_a_ = 68.158 − 18.425FB + 1.266FB ^2^ − 0.276HL + 0.001HL^2^ + 0.038FB∙HL.

Graphic representation of the variation in air void content in AC 8 S as a function of the amount of foamed bitumen and hydrated lime is shown in Figure 5. 

Analysis of the results presented in Figure 5 confirms that as the amount of foamed bitumen and hydrated lime increases, the air void content in the asphalt concrete decreases throughout the experiment. At the same time, hydrated lime has a significant impact on the assessed parameter in the range from 5.6% to 5.9% of 50/70 foamed bitumen as it contributes to an increase in air voids; its recommended content (max. 4.0%) is even exceeded. The increase in foamed bitumen content in the range from 5.9% to 6.2% has a positive effect on this parameter and enables it to obtain the recommended values [58]. A further increase in foamed bitumen content to 6.5% results in a significant air voids reduction to the level below the required level. [58]. 

### 3.2. The Effects of the Foamed Bitumen and Hydrated Lime on Moisture Resistance of Asphalt Concrete

In accordance with the adopted research plan, the assessment of moisture and frost resistance of the AC 8 S mixture with foamed bitumen and hydrated lime was based on the WT-2 2014 procedure [58] and on the modified AASHTO T283 procedure [50,51]. The test methodology according to the modified AASHTO T283 method is based on a fairly aggressive asphalt concrete conditioning scheme, which is to simulate very variable and stringent climatic conditions. 

#### 3.2.1. Water Sensitivity According to WT-2 2014

The procedure for assessing water sensitivity of an asphalt mix according to the requirements of WT-2 2014 [58] has only one freeze-thaw cycle on water-saturated specimens. The basis for the assessment is the ITSR, which should be greater than 90% for the bituminous mixture designed for the wearing course to be resistant to moisture and frost. When the mixture is characterized by high fineness and rather high binder content, this ratio reaches a value greater than 100% [58,64].

**Figure 6:**
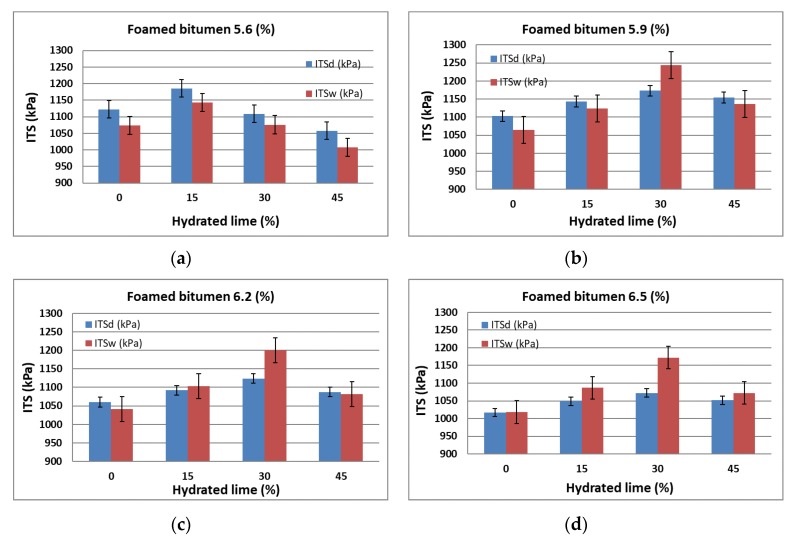
Indirect tensile strength ITS_d_ and ITS_w_ of AC 8 S versus the contents of hydrated lime and 0.6% SAA modified foamed bitumen at: (**a**) 5.6 %, (**b**) 5.9%, (**c**) 6.2%, (**d**) 6.5%.

In the first stage of AC 8 moisture and frost resistance assessment, first the indirect tensile strength ITS_d_ for unconditioned specimens and then ITS_w_ for moisture-conditioned specimens were determined to WT-2 2014 [58]. The test results are summarized in Table 7 and graphically represented in Figure 6.

An increase in the 50/70-grade foamed bitumen content in the AC 8 S mixture lowers ITS_d_ and ITS_w_ values, which is in line with the general trend in this respect. The use of hydrated lime increases ITS_d_ and ITS_w_. With 5.6% binder in the mixture, this relationship only occurs with the addition of 15% hydrated lime. When its content increases, the value of the analyzed parameter decreases. This may be due to the high concentration of hydrated lime and a small amount of binder, which stiffens the AC 8 S mixture. Higher hydrated lime content tends to increase ITS_d_ and ITS_w_. This trend increases with the amount of hydrated lime increased to 30%. It is most likely the effect of a favorable improvement of the binder adhesion to the aggregate and its interaction as a mineral lime filler with very small particle size [61]. Increasing the hydrated lime content to 45% has a negative effect as a result of introducing too much fine-grained material and insufficient amount of binder to coat it, leading to excessive stiffening of the mixture. Attention should be paid to adsorption at the interface between the strongly basic mineral material (hydrated lime) and the binder, which contributes to a decrease in the content of "free binder" in the structure of the mixture [61]. This further increases its rigidity and lowers the values of ITS_d_ and ITS_w_ [40].

Overall, with the binder content of 5.9% and hydrated lime content of 30% in the AC 8 S HWMA mixture, the indirect tensile strength ITS_d_ and ITS_w_ met the normative requirements of WT-2 2014 [58] most favorably.

To comprehensively describe the relationship of indirect tensile strength ITS_d_ and ITS_w_ of the AC 8 HWMA mixture with foamed bitumen 50/70, 0.6% SAA and hydrated lime, the second-degree polynomial model was adopted. In the first stage of model assessment, the significance test was performed using ANOVA [63] (Table 8).

Analysis of the parameters listed in Table 8 indicates clearly that foamed bitumen and hydrated lime constitute a factor that has a significant effect on the indirect tensile strength, ITS_d_ and ITS_w_, of the AC 8 S, as demonstrated by *p*-values being less than the pre-defined significance level α = 0.05. Statistical significance was not observed in the assessment of linear effects of hydrated lime on ITS_d_ and foamed bitumen on ITS_w_. Interactions found between the foamed bitumen and hydrated lime contents also affect the value of this parameter (*p*-value less than α = 0.05).

The parameters describing the regression model for ITS_d_ and ITS_w_ of AC 8 S as a function of the amounts of foamed bitumen and hydrated lime are given in Table 9. 

The adjusted coefficient of determination R^2^ of the model for ITS_d_ and ITS_w_ is nearly 53%, which confirms the adequacy of the model. Both the amount of foamed bitumen 50/70 alone and the amount of hydrated lime alone have a significant impact on the flexural tensile strength, ITS_d_ and ITS_w_ of the AC 8 HWMA mixture. An interaction of these factors is observed (*p*-value is less than the pre-defined significance level α = 0.05). 

**Figure 7:**
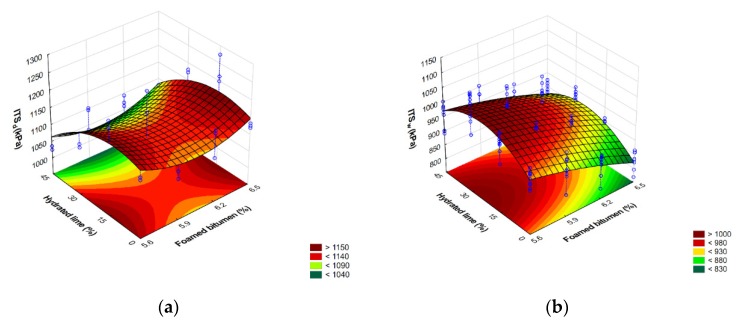
Flexural indirect tensile strength (**a**) ITS_d_ and (**b**) ITS_w_ of AC 8 S versus foamed bitumen and hydrated lime contents; representation of the models describing these relationships. ITS_d_ = −4950.47 + 2144.17FB − 189.18FB^2^ − 11.18HL − 0.08HL^2^ + 2.50FB∙HL. ITS_w_ = −7271.46 + 2828.03FB − 239.57FB^2^ − 10.08HL − 0.18HL^2^ + 3.16FB∙HL.

Graphic representation of the variation in the ITS_d_ and ITS_w_ values of the AC 8 S mixture in terms of foamed bitumen and hydrated lime is shown in Figure 7. 

Analysis of the results presented in Figure 6 confirms that with an increase in the amount of foamed bitumen and hydrated lime, the value of dry tensile strength ITS_d_ as per WT-2 2014 [58] reaches a maximum at a 6.2% bitumen content in the mixture. At the same time, hydrated lime has a significant impact on the ITS_d_ in virtually the entire dosing range.

A comprehensive analysis of the results based on the assessment of the relationship presented in Figure 7 shows that as the amounts of foamed bitumen increase and at hydrated lime increase from 15% to 30%, the wet tensile strength ITS_w_ of the specimens conditioned as per WT-2 2014 increases significantly [58]. Please note the synergy of hydrated lime and foamed bitumen that significantly affect the ITS_w_ of the mixture.

Assessment of the resistance of the AC 8 S mixture to climatic factors according to WT-2 2014 is based on the ITSR expressed as the ratio of the tensile strength of moisture-conditioned specimen to the tensile strength of air-conditioned specimen (ITS_w_/ITS_d_). 

The methodology developed as per WT-2 2014 assumes that the AC 8 S bituminous mixture is resistant to climatic factors (water and frost) when the ITSR reaches at least 90% [58].

**Figure 8:**
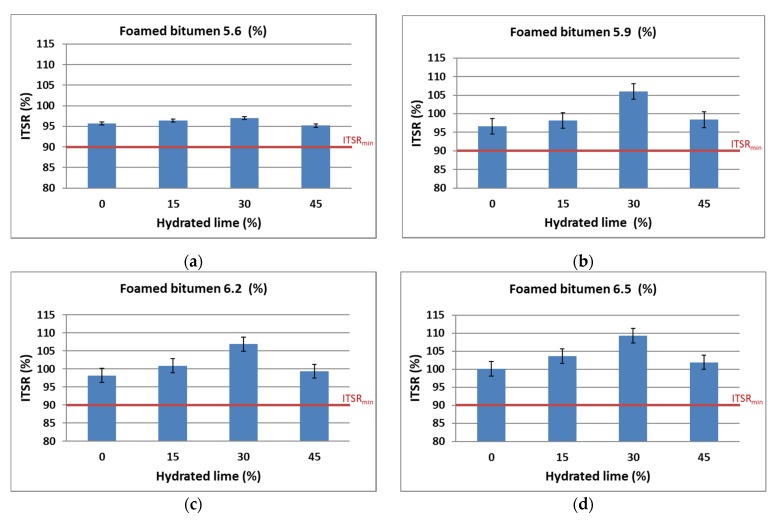
The ITSR of AC 8 S versus the contents of hydrated lime and 0.6% SAA modified foamed bitumen at: (**a**) 5.6%, (**b**) 5.9%, (**c**) 6.2%, (**d**) 6.5%.

Graphic representation of the ITSR as a function of technology used, the amount of binder, and the hydrated lime content is shown in Figure 8. 

Analysis of the test results indicates that the AC 8 S mixture is resistant to moisture and frost in the entire range of use of 50/70-grade foamed bitumen with 0.6% SAA and hydrated lime—the ITSR always reaches values higher than 90%. The variation in its value depending on the constituents used (foamed bitumen, hydrated lime) is analogous to that of ITS_d_ and ITS_w_.

Overall, it was observed that with 5.9% binder and 30% hydrated lime in the filler, the AC 8 S asphalt concrete has obtained the greatest increase in the water sensitivity described by the ITSR. The mixtures with higher binder contents have yielded slightly higher ITSR results; however, the high bitumen content could be a concern as for the resistance to permanent deformation performance and cost of such mixtures.

The relationship between the moisture resistance of the AC 8 HWMA mixture with 0.6% SAA modified 50/70-grade foamed bitumen and hydrated lime was described using the second-degree polynomial model. In the first stage in the model assessment, the significance test was performed using ANOVA variance analysis [63] (Table 10).

Analysis of the parameters listed in Table 10 indicates clearly that foamed bitumen and hydrated lime contents constitute a factor that has a significant effect on the indirect tensile strength ratio ITSR in the AC 8 S, as demonstrated by the *p*-values being less than the pre-defined significance level α = 0.05. No statistical significance was observed only in the case of the quadratic component of the model, related to the bitumen amount, and of the interaction term. 

The parameters describing the regression model of the ITSR of the AC 8 S as a function of foamed bitumen and hydrated lime contents are given in Table 11. 

The adjusted coefficient of determination R^2^ is nearly 58%, which indicates the adequacy of the model. Hydrated lime has a significant effect on the value of ITSR for the AC 8 S HWMA mixture (*p*-value is less than the pre-defined significance level α = 0.05). A deviation from the presented trend exists for the linear relationship between the lime and the linear and squared terms related to the foamed bitumen content in the mixture. 

**Figure 9:**
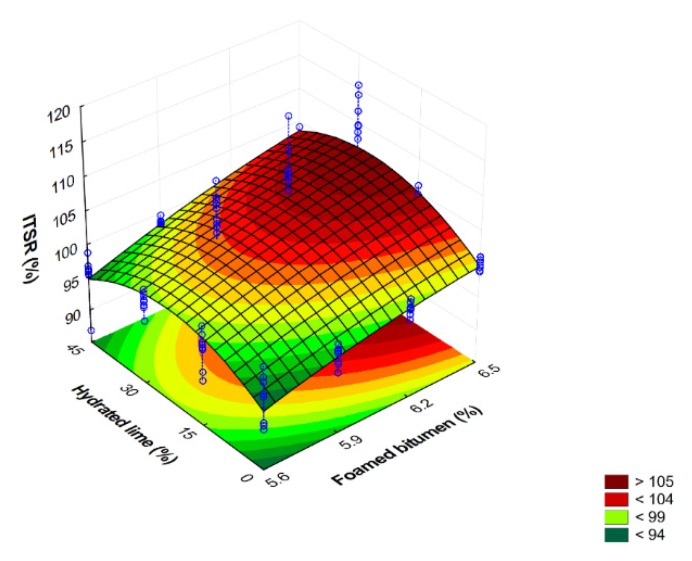
Water sensitivity–ITSR of AC 8 S in terms of foamed bitumen and hydrated lime contents, and the representation of the model describing this relationship. ITSR = −77.012 + 51.119FB − 3.684FB^2^ − 0.031HL − 0.009HL^2^ + 0.072FB∙HL.

Graphic representation of the variation in the ITSR value of the AC 8 S mixture in terms of the contents of foamed bitumen-grade 50/70 and hydrated lime is shown in Figure 9. 

Comprehensive analysis of the results based on the relationships shown in Figure 9 indicates clearly that the contents of foamed bitumen increasing from 5.9% to 6.5% and hydrated lime increasing from 0% do 30% have a significant effect of increased ITSR, which corresponds to the resistance of the AC 8 S mixture to water and frost as per WT-2 2014 [58]. An interaction effect between the hydrated lime and foamed bitumen provides the mixture with water and frost resistance.

**Figure 10:**
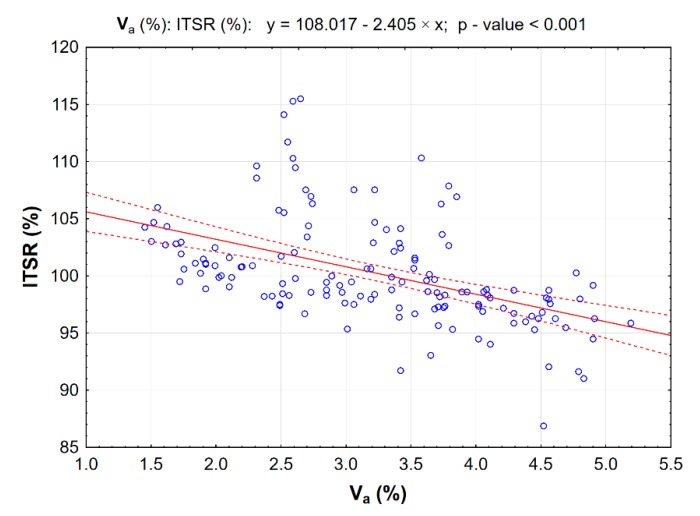
Correlation between AC 8 S mixture air void content V_a_ and ITSR; dotted lines represent 95% confidence interval.

At the same time, it is important to determine the correlation between the air void content V_a_ and ITSR (Figure 10).

The correlation presented is linear and statistically significant, as the *p*-value is less than the pre-defined significance level α = 0.05. 

#### 3.2.2. Resistance to Water Damage According to the Modified AASHTO T283 Method

The procedure of assessing moisture and frost resistance according to the modified AASHTO T283 method consists of subjecting specimens of the bituminous mixture to 18 freeze-thaw cycles. In comparison with WT-2 (one freezing cycle), this procedure is more severe in terms of exposure of asphalt concrete to climatic factors. The simulation of the in-service conditions is closer to natural conditions. Thus, the results of moisture and frost resistance tests according to the modified AASHTO T283 method represent the behavior of the mixture in real conditions better than those obtained from the WT-2 2014 procedure. 

The moisture-induced damage resistance (AASHTO T283) is reflected in the value of RW_wm_, which is defined as the indirect tensile strength of moisture-conditioned specimens divided by the indirect tensile strength of dry specimens.

**Figure 11:**
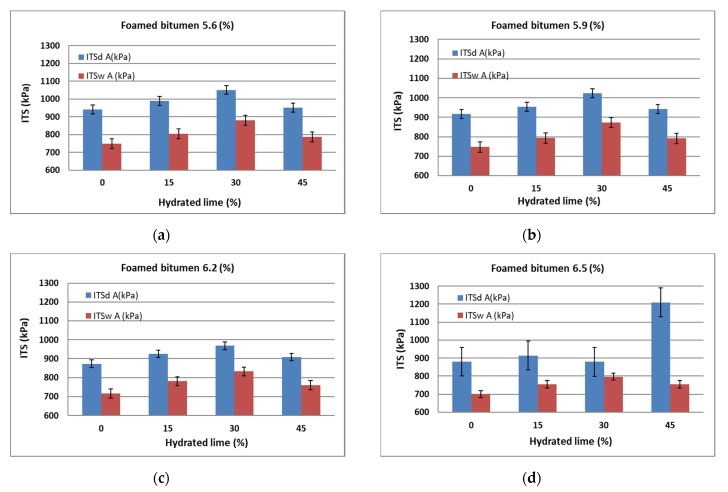
Relationship between the indirect tensile strength of dry specimens ITS_d_^A^ and moisture-conditioned specimens ITS_w_^A^ (modified AASHTO T283) of the AC 8 S mixture versus the technology used, amount of hydrated lime and foamed bitumen: (**a**) 5.6%, (**b**) 5.9%, (c) 6.2%, (**d**) 6.5%.

In the first step of the assessment of AC 8 S moisture e resistance as per AASHTO T283, indirect tensile strength tests for dry (ITS_d_^A^) and moisture-conditioned specimens (ITS_w_^A^) were performed, as shown in Table 12. Graphic representation of the results is in Figure 11. 

Analysis of the results of flexural tensile strength ITS_d_^A^ for the dry-conditioned AC 8 S mixture indicates that hydrated lime up to 30% increases the analyzed parameter for the HWMA mixture. Increasing its content to 45% leads to a decrease in ITS_d_^A^. With increasing concentration of the binder in the mixture, the value of the analyzed parameter decreases, which is consistent with the observations made so far [33]. 

It should also be noted that the presented trend of dry indirect tensile strength (modified AASHTO T283) ITS_d_^A^ for the AC 8 S in terms of the applied technology, amount of binder, and hydrated lime content has the same character as that obtained from the WT-2 2014 procedure [58].

It can thus be concluded that a favorable flexural tensile strength was obtained with the 5.9% binder content (bitumen 50/70 + 0.6% SAA by weight) and 30% concentration of hydrated lime in the filler added to the AC 8 S HWMA mixture. 

Analysis of the results of the effect of 50/70-grade bitumen content with 0.6% SAA and hydrated lime on the flexural indirect tensile strength ITS_w_^A^ of the moisture-conditioned mixture indicates that they exhibit the same trend as those from “dry” conditioned specimens.

Overall, the binder content of 5.9% and hydrated lime content of 30% in the AC 8 S HWMA mixture resulted in a favorable flexural tensile strength. 

Comprehensive description of the relationships of ITS_w_^A^ of the AC 8 S HWMA mixture with foamed 50/70 bitumen, 0.6% SAA addition and hydrated lime was prepared using the second-degree polynomial model. The first stage of the model evaluation involved performing a significance test using the ANOVA [63] (Table 13).

Analysis of the parameters compiled in Table 13 shows that the contents of foamed bitumen and hydrated lime constitute a significant factor affecting the indirect tensile strength ITS_d_^A^ and ITS_w_^A^ of the AC 8 S, as the *p*-values are less than the pre-defined significance level α = 0.05. No statistical significance was observed for the quadratic terms related to the binder effect. 

No interaction effects were found between the foamed bitumen content and hydrated lime content. This can be attributed to the stringent conditioning of the specimens as a result of which this factor did not show a significant effect on the parameter. Consequently, statistically significant synergy of the hydrated lime and foamed bitumen did not occur. 

The values describing the parameters of the regression model of the response surface for the indirect tensile strength ITS_d_^A^ and ITS_w_^A^ of the AC 8 S mixture in terms of the foamed bitumen and hydrated lime contents are summarized in Table 14.

The adjusted coefficient of determination R^2^ for ITS_d_^A^ is nearly 62% and 60% for ITS_w_^A^, which indicates the adequacy of the models. Hydrated lime has a significant influence on the dry indirect tensile strength ITS_d_^A^ of the AC 8 S HWMA mixture (*p*-value is less than the pre-defined significance level α = 0.05). 

Hydrated lime (*p*-value is less than the pre-defined significance level α = 0.05) has a statistically significant effect on the indirect tensile strength ITS_w_^A^ of the AC 8 S HWMA mixture. However, no effect of the foamed bitumen content and no interaction between the contents of bitumen and hydrated lime were observed, which seems to be a consequence of rigorous conditions of specimen conditioning in the freeze-thaw process.

**Figure 12:**
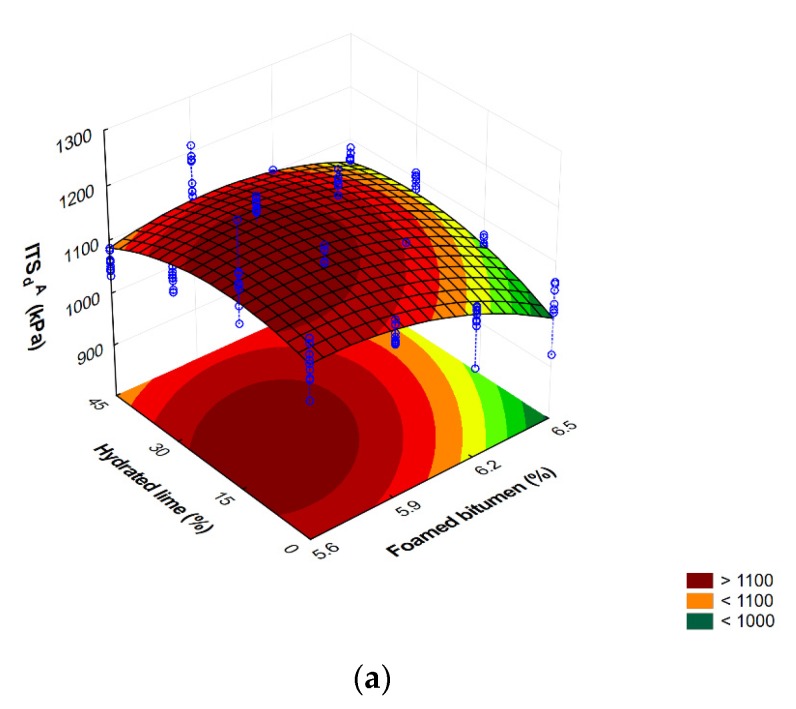

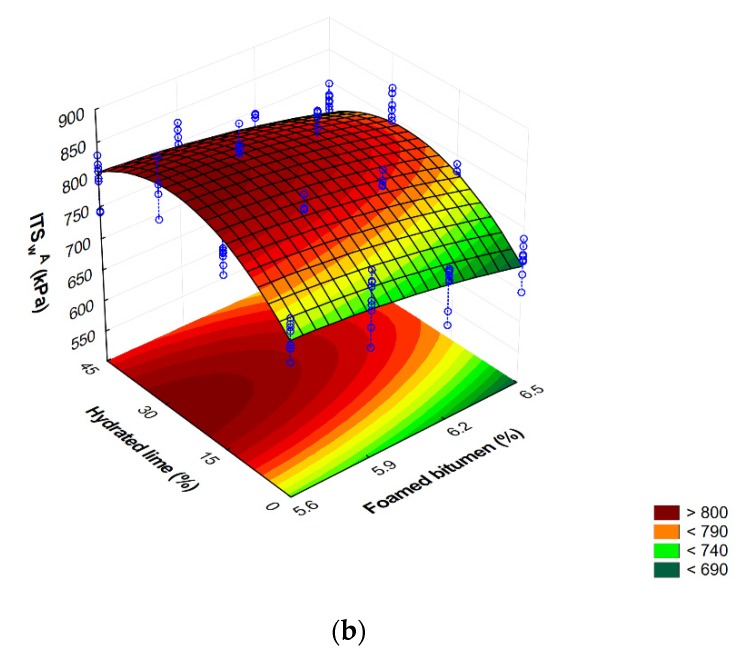
Indirect tensile strength after dry conditioning ITS (**a**) and freeze-thaw protocol as per modified AASHTO T283 (**b**) of AC 8 S mixture versus the contents of foamed 50/70-grade bitumen and hydrated lime and the representation of the model describing this relationship. ITS_d_^A^ = −228.459 + 502.201FB − 52.446FB^2^ + 3.410HL − 0.128HL^2^ + 0.545FB∙HL. ITS_w_^A^ = −605.351 + 502.984FB − 46.752FB^2^ + 7.572HL − 0.142HL^2^ + 0.020FB∙HL.

Graphic representation of the variation in the flexural indirect tensile strength ITS_d_^A^ and ITS_w_^A^ of the AC 8 S mixture versus the contents of foamed 50/70-grade bitumen and hydrated lime is shown in Figure 12. 

Comprehensive analysis of the results based on the response surface shown in Figure 12 indicates that increasing the content of foamed bitumen and the content of hydrated lime from 15% to 30% has a significant effect on the increase in wet indirect tensile strength ITS_w_^A^ of the AC 8 S mixture (the modified AASHTO T283 procedure).

**Figure 13:**
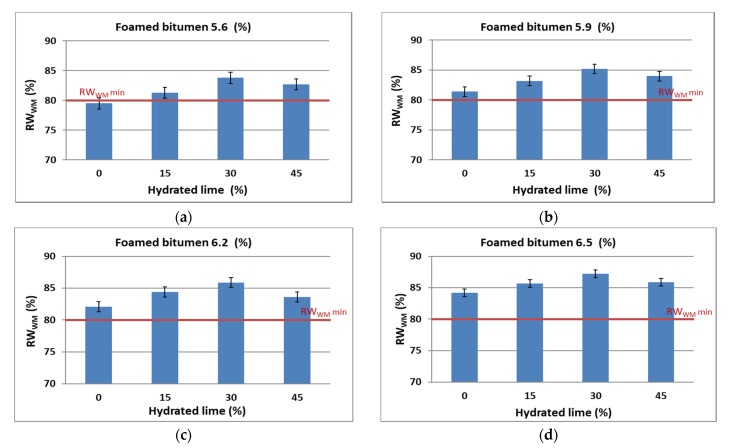
Relationship of the moisture and frost resistance—RW_WM_ according to the modified AASHTO T283 method for AC 8 S in terms of the technology type and contents of hydrated lime and foamed bitumen: (**a**) 5.6%, (**b**) 5.9%, (**c**) 6.2%, (**d**) 6.5%.

The resistance of the AC 8 S mixture to climatic factors as per AASHTO T283 is assessed based on the RW_WM_, which is an ITS_w_^A^/ITS_d_^A^ ratio. Calculation results are shown graphically in Figure 13.

According to the methodology developed based on the modified AASHTO T283 procedure, a bituminous mixture is resistant to climatic factors—moisture and frost—when the RW_WM_ is at least 80%.

Analysis of the results shows that increasing content of the binder in the AC 8 S mixture increases the value of RW_WM_, hence, the resistance to moisture damage. This response was expected and compliant with the general principles of asphaltic materials technology. The use of hydrated lime increases the value of this parameter and at 30% lime in the limestone dust, the resistance to moisture, and frost satisfies the requirements. A further increase in the lime content decreases the value of this parameter which can be attributed to a significant increase in the specific area of the filler (mineral dust + hydrated lime) in relation to the binder content.

A comprehensive description of moisture and frost resistance of the AC 8 S HWMA mixture with foamed bitumen, 0.6% SAA and hydrated lime was prepared using the second-degree polynomial model. The first stage of the model evaluation included a significance test using ANOVA variance analysis (Table 15).

Analysis of the parameters presented in Table 15 shows that the contents of foamed bitumen and hydrated lime constituted a significant factor that influenced the moisture and frost resistance (RW_WM_ ) of the AC 8 S mixture, as demonstrated by the *p*-values being less than the pre-defined significance level α = 0.05. This relationship was not observed only for the square term describing the influence of bitumen content. The existence of interaction between the content of foamed bitumen and hydrated lime, which influence the analyzed parameter (*p*-value is less than α = 0.05), is important, as it supports the use of foamed bitumen and hydrated lime in the mixture.

The value of the adjusted R^2^ is almost 76%, which indicates the adequacy of the adopted model. The parameters of the developed regression model of the relationship between the RW_WM_ of the AC 8 S mixture and the foamed bitumen and hydrated lime contents are presented in Table 16.

Analysis of the parameters listed in Table 16 shows that hydrated lime is a significant factor influencing the resistance of the AC 8 S mixture to moisture, characterized by the RW_WM_, as the *p*-value is less than the pre-defined significance level α = 0. An interaction exists of the influence of foamed asphalt and hydrated lime with respect to the RW_WM_ (*p*-value less then α = 0.05) as per the modified AASHTO T283 method.

**Figure 14:**
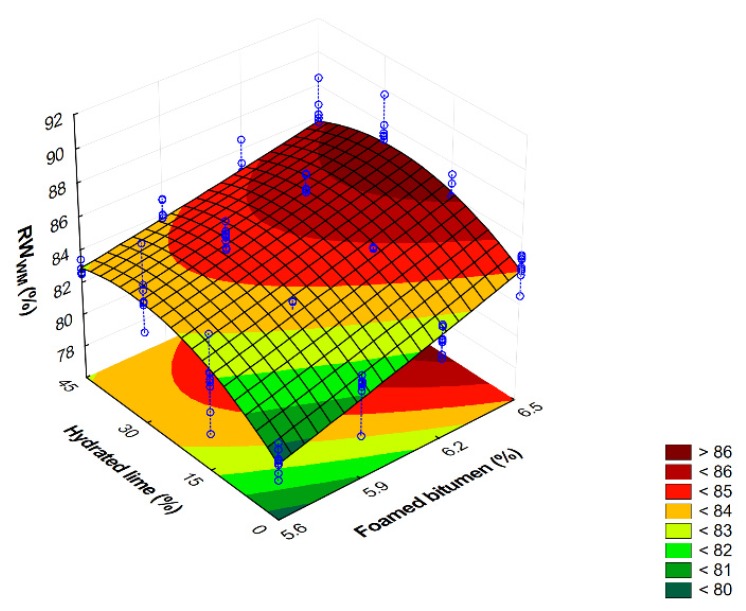
Relationship of RW_WM_ (modified AASHTO T283) of AC 8 S in terms of foamed bitumen and hydrated lime contents. RW_WM_ = 63.799 + 0.774*FB* + 0.359*FB*^2^ + 0.485HL − 0.003*HL*^2^ – 0.043*FB∙HL.*

Graphic representation of moisture and frost resistance relationship model—RW_WM_ (AASHTO T283) of the AC 8 S HWMA mixture in terms of foamed asphalt and hydrated lime contents is presented in Figure 14 together with the developed model.

Comprehensive analysis of the results based on the relationship shown in Figure 12 indicates that the contents of foamed bitumen increasing from 5.9% to 6.2% and hydrated lime from 15% to 30% have a significant effect on the RW_WM_ increase, which corresponds to the resistance to moisture and frost (modified AASHTO T283). Hydrated lime and foamed bitumen interact to provide resistance to moisture-induced damage.

**Figure 15:**
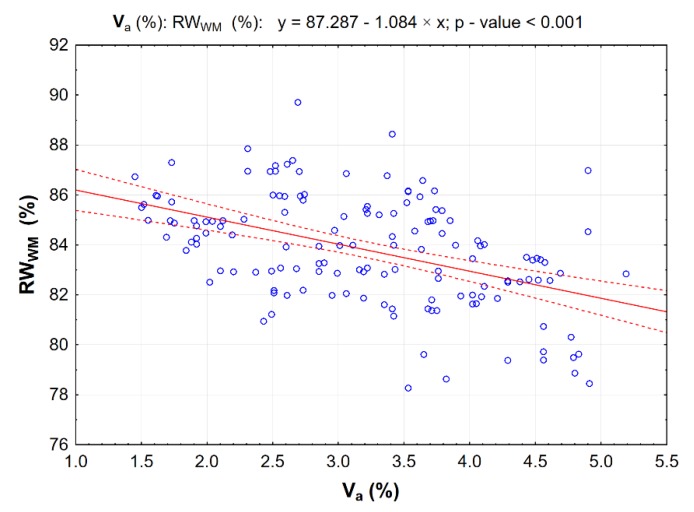
Correlation between air void content V_a_ and moisture resistance as per modified AASHTO T283 (RW_WM_) in the AC 8 S bituminous mixture; dotted lines represent 95% confidence interval.

To assess the effect of hydrated lime and 50/70-grade foamed bitumen with an addition of 0.6% SAA on the properties of the AC 8 S bituminous mixture, the correlation between the air void content V_a_ and resistance to water and frost RW_WM_ (Figure 15) was analyzed. 

The statistically significant correlation between the air void content V_a_ of AC 8 S and its resistance to water and frost (RW_WM_) has a linear character (*p*-value less than α = 0.05). 

### 3.3. Optimization of the Foamed Bitumen and Hydrated Lime Contents in the AC 8 S Mixture in Terms of its Resistance to Moisture and Frost 

To determine the recommended amount of foamed asphalt and hydrated lime to obtain the most favorable parameters characterizing the resistance of AC 8 S to moisture and frost, the following parameters were analyzed:

- air void content (V_a_),
- water sensitivity to WT-2 (ITSR),
- moisture-induced damage resistance to modified AASHTO T283 method (RW_WM_).

Analysis of the relationships between the evaluated properties of asphalt concrete AC 8 S is an important element in the assessment of the effects of hydrated lime and 50/70-grade foamed bitumen with the addition of 0.6% SAA. Correlations between these properties are summarized in Table 17.

The main correlation parameter was the air void content V_a_, which has a significant effect on other properties of AC 8 S. The correlation values represent most of the results obtained in this experiment. From the results it follows that they are mostly non-linear or that statistically significant interactions exist.

The characteristics of the models describing the analyzed relationships of the AC 8 S parameters in terms of the contents of 50/70-grade foamed bitumen and hydrated lime are presented in Table 18. 

To assess the performance of AC 8 S, criteria were adopted, according to which the most desirable values of the parameters were assigned the performance indicator equal to 1 and the values least desired: 0. Intermediate values obtained indicators from the range 0 to 1 in the linear relationship. The used optimization procedure has been described in detail in [65]. The following criteria were applied for individual parameters of asphalt concrete:

Air void content V_a_ (max: 0, min: 1),

ITSR according to WT-2 (max: 1, min: 0),

RW_WM_ according to the modified AASHTO T283 method (max: 1, min: 0).

**Figure 16:**
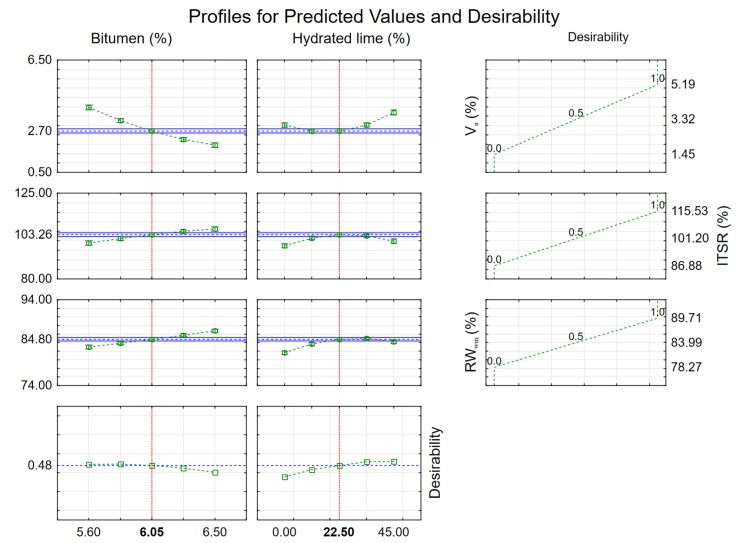
Optimum contents of 50/70-grade bitumen and hydrated lime to ensure water and frost resistance of AC 8 S.

Then the values of the utility function of the asphalt concrete were calculated. The approximated results are plotted in Figure 16.

On the basis of the utility analysis of the approximated values it was found that in order to ensure the moisture and frost resistance of asphalt concrete AC 8 S, the recommended content of 50/70 foamed bitumen with 0.6 % SAA should be 6.05 % and that of hydrated lime in the mineral filler 22.5 %. 

## 4. Conclusion

The following conclusions were drawn based on the moisture resistance tests of AC 8 S HWMA mixture:

- The use of 50/70-grade foamed bitumen with an addition of 0.6% SAA and hydrated lime in the AC 8 S mixture has a significant effect on its properties. The intensity of the effects of these constituents varies by parameter.
- Hydrated lime used as a replacement for limestone dust is important for air void content. With up to 30%, it has a beneficial effect on the air void content regardless of the foamed bitumen content in the asphalt concrete. When it is used at more than 30%, the trend changes, which can be attributed to the fact that at higher concentrations hydrated lime impedes compaction due to higher demand for bitumen as a result of a larger specific area compared to that of the mineral dust.
- Similar relationship is observed in the case of the resistance to moisture and frost, ITSR, determined to WT-2 2014. The interaction between the contents of hydrated lime and foamed bitumen plays a role here as they influence this parameter of the mixture.
- Moisture-induced damage test to the modified AASHTO T283 method indicates that 15% and more hydrated lime used in the mixture composition provides optimum values of this characteristic. There is also an interaction between the contents of hydrated lime and binder, with a significant effect on the resistance of the mixture to water and frost (the modified AASHTO T283 method).
- The authors found the synergy of the hydrated lime and foamed bitumen, depending on their contents in the asphalt concrete, for ensuring the resistance of the AC 8 S HWMA mixture to moisture and frost.
- Regardless of the research methods used, resistance to moisture and frost can be ensured by optimizing the contents of foamed bitumen and hydrated lime in the HWMA mixture.
- Optimization of the AC 8 S HWMA mixture in terms of the evaluated parameters determined the contents of hydrated lime and foamed bitumen at, considering the dosing tolerance, 30% and 5.9. The in-service resistance of the pavement wearing course to moisture and frost will be ensured.

The conducted research has proven that correct mixture design permits superior moisture and frost resistance of the tested AC 8 S HWMA with foamed bitumen. It was shown that incorporation of hydrated lime as a significant part of the filler fraction (30%) strongly contributed to the performance of the HWMA mixture when optimum amount of foamed bitumen was used. Despite the high surface area of the hydrated lime requiring increased amounts of bitumen, the hydrated lime bearing HWMA mixtures exhibited adequate volumetric performance and increased resistance to frost and moisture damage tested by the means of different methods. The incorporation of hydrated lime has proven to be a feasible method for ensuring the adequate performance and durability of the AC 8 S HWMA mixture with foamed bitumen.

## Figures and Tables

**Table 1:** Characteristics of the surface-active agent SAA.

Property	Unit of Measure	Value
Appearance	-	Brown viscous liquid
Density at 20 °C	Mg/m^3^	0.98
Pour point	°C	<0
Viscosity at 20 °C	mP	3000
Viscosity at 50 °C	mP	400
Amine index	mg HCl/g	159–185
Acid index	mg KOH/g	<10
Freezing point	°C	<0
Flash point (open flame)	°C	>218

**Table 2:** Properties of neat 50/70 and bitumen with 0.6% SAA content.

Property	Unit	Testing Method	Bitumen	
			50/70	50/70 + 0.6% SAA
Penetration at 25°C	0.1 mm	PN-EN 1426	65.9	70.4
Softening point T_R&B_	°C	PN-EN 1427	50.4	48.8
Fraass breaking point	°C	PN-EN 12593	−15.1	−14.2
Temperature plasticity range	°C	-	65.5	63.0
Penetration Index	-	EN 12591	−0.6	1.4
Expansion ratio ER	-	[19,29]	11	19
Half-life HL	s		10	21
Foaming water content FWC	%	-	2.5	2.5

**Table 3:** Composition of AC 8 mineral mixture.

Materials	Mineral Mixture (% m/m)	Bituminous Mixture (% m/m)
Filler (limestone aggregate)	7.0	6.6
Crushed fine continuously graded aggregate 0/2 mm (limestone)	37.0	34.8
Coarse aggregate 2/5 mm (gabbro)	16.0	15.1
Coarse aggregate 4/8 mm (gabbro)	40.0	37.7
50/70 penetration paving-grade bitumen	-	5.6
Total	100.0	100.0

**Table 4:** Statistical values of air void content V_a_ asphalt concrete AC 8 S.

Variables		Value of Statistical Parameters of 50/70 Bitumen Properties				
Foamed Bitumen with 0.6 SAA (%)	Hydrated Lime (%)	Max.	Min.	Mean	Std. Dev.	Coef. Var. (%)
5.6	0	4.91	4.29	4.67	0.19	4.17
	15	3.93	3.06	3.49	0.24	7.00
	30	4.90	4.02	4.43	0.34	7.77
	45	5.21	4.29	4.54	0.27	6.08
5.9	0	4.21	3.69	3.94	0.19	4.90
	15	3.35	2.56	2.95	0.21	7.25
	30	3.79	3.22	3.58	0.26	7.26
	45	4.11	3.63	3.81	0.17	4.57
6.2	0	2.73	2.37	2.53	0.11	4.58
	15	2.28	1.90	2.09	0.12	5.80
	30	3.06	2.48	2.68	0.16	6.07
	45	3.64	2.76	3.18	0.23	7.42
6.5	0	2.11	1.72	1.89	0.11	6.30
	15	1.83	1.45	1.60	0.10	6.39
	30	2.74	2.31	2.51	0.13	5.31
	45	3.62	3.21	3.44	0.12	3.46

**Table 5:** Statistical significance of the effects of foamed bitumen with SAA and hydrated lime on air void content in AC 8 S (ANOVA).

Effect	Variable: Va (%); R^2^ = 0.880; R^2^ -adj = 0.875; Pure Error MS = 0.040			
	SS	MS	F	*p*-Value
(1) Foam. bitum. (%)(L)	81.115	81.115	2018.839	<0.001
Foam. bitum. (%)(Q)	1.870	1.870	46.537	<0.001
(2) Hydr. lime (%)(L)	8.975	8.975	223.377	<0.001
Hydr. Lime (%)(Q)	12.325	12.325	306.760	<0.001
1L× 2L	6.776	6.7760	168.633	<0.001
Lack of fit	10.025	1.003	24.950	<0.001
Pure error	5.143	0.040	-	-
Total SS	126.229	-	-	-

SS—Sum of Squares, MS—Mean Squares; F—Fisher statistic, red font denotes statistical significance with *p*-value greater than pre-defined significance level α = 0.05.

**Table 6:** Parameters of the model of the relationship between V_a_ of AC 8 S and the amounts of foamed bitumen with 0.6% SAA and hydrated lime.

Response	Effect	Regression Coeff.	SE	T(128)	*p*-Value	–95% Cnf. Lmt	+95% Cnf. Lmt
*V_a_* R^2^ = 0.880	Intercept	68.159	6.791	10.036	<0.001	54.720	81.596
	(1) Foamed bitum. (%) (L)	−18.425	2.247	−8.199	<0.001	−22.872	−1.978
	Foamed Bitum. (%) (Q)	1.266	0.185	6.822	<0.001	0.898	1.6334
	(2) Hydrated lime (%) (L)	−0.276	0.018	−15.132	<0.001	−0.134	−0.241
	Hydrated lime (%) (Q)	0.001	0.001	17.515	<0.001	0.001	0.001
	1L × 2L	0.038	0.003	12.986	<0.001	0.032	0.044

**Table 7:** Values of ITS_d_ and ITS_w_ (WT-2 2014) for AC 8 S mixture; SD—standard deviation, CV—coefficient of variation.

Variables		ITS_d_ (kPa)			ITS_w_ (kPa)		
Foamed Bitumen with 0.6% SAA (%)	Hydrated Lime (%)	Mean	SD	CV (%)	mean	SD	CV (%)
5.6	0	1122.4	34.63	3.08	1074.0	20.27	1.88
	15	1185.7	49.96	4.21	1143.1	35.47	3.10
	30	1109.1	14.79	1.33	1076.1	26.03	2.41
	45	1058.0	17.44	1.64	1007.9	30.89	3.06
5.9	0	1102.6	16.07	1.45	1065.3	24.64	2.31
	15	1143.6	29.49	2.57	1124.3	38.34	3.41
	30	1173.1	18.87	1.60	1243.9	42.73	3.43
	45	1154.1	38.73	3.35	1136.5	36.91	3.24
6.2	0	1060.4	35.87	3.38	1041.7	31.39	3.01
	15	1091.9	34.30	3.14	1102.8	36.24	3.28
	30	1124.1	51.05	4.54	1200.2	34.08	2.83
	45	1087.8	12.27	1.12	1081.6	14.20	1.31
6.5	0	1016.9	41.12	4.04	1018.6	43.02	4.22
	15	1048.8	13.28	1.26	1086.9	10.38	0.95
	30	1072.6	48.51	4.52	1172.0	59.15	5.04
	45	1051.7	35.98	3.42	1072.6	37.84	3.52

**Table 8:** Evaluation of the effect of foamed bitumen with 0.6% SAA and hydrated lime in asphalt concrete on ITS_d_ and ITS_w._

**Effect**	**Variable: ITS_d_ (kPa); ANOVA; R^2^ = 0.545; R^2^ -adj : 0.529, MS = 1121.321**			
	**SS**	**MS**	**F**	***p*-Value**
(1) Foamed bit. (%) (L)	127,467.223	127,467.223	113.675	<0.001
Foamed bit. (%) (Q)	41,745.336	41,745.336	37.228	<0.001
(2) Hydrated Lime (%) (L)	2775.453	2775.453	2.475	0.118
Hydrated Lime (%) (Q)	48,848.483	48,848.483	43.563	<0.001
1L × 2L	28,460.817	28,460.817	25.381	<0.001
Error	64,199.922	6420.002	5.725	<0.001
Total SS	143,529.151	1121.357		
**Effect**	**Variable: ITS_w_ (kPa); ANOVA; R^2^ = 0.539; Adj. R^2^: 0.523, MS = 1195.067**			
	**SS**	**MS**	**F**	***p*-Value**
(1) Foamed bit.(%) (L)	1.511	1.511	0.001	0.972
Foamed bit. (%) (Q)	669,42.024	66,942.024	56.016	< 0.001
(2) Hydrated Lime (%) (L)	31,816.351	31,816.351	26.623	< 0.001
Hydrated Lime (%) (Q)	238,436.924	238,436.924	199.517	< 0.001
1L × 2L	45,424.441	45,424.441	38.010	< 0.001
Error	173,549.862	17,355.018	14.522	< 0.001
Total SS	152,968.547	1195.131		

**Table 9:** Parameters of the model of the relationship between ITS_d_, ITS_w_, and the amounts of foamed bitumen with 0.6% SAA and hydrated lime.

Response	Effect	Parameter	SE	t(18)	*p*-Value	−95% Cnf. Lmt	+95% Cnf. Lmt
*ITS_d_* R^2^ = 0.545	Intercept	−4950.472	1134.541	−4.363	<0.001	−7195.354	−2705.591
	(1)Foamed bit. (%)(L)	2144.175	375.427	5.711	<0.001	1401.326	2887.012
	Foamed bit. (%)(Q)	−189.183	31.006	−6.101	<0.001	−250.532	−127.834
	(2)Hydrated lime (%)(L)	−11.187	3.057	−3.655	<0.001	−17.225	−5.135
	Hydrated lime (%)(Q)	−0.083	0.012	−6.600	<0.001	−0.111	−0.061
	1L × 2L	2.501	0.496	5.038	<0.001	1.527	3.485
*ITS_w_* R^2^ = 0.539	Intercept	−7271.461	1171.254	−6.208	<0.001	−9588.991	−4953.935
	(1)Foamed bit. (%)(L)	2828.033	387.576	7.296	<0.001	2061.142	3594.914
	Foamed bit. (%)(Q)	−239.576	32.009	−7.484	<0.001	−302.905	−176.233
	(2)Hydrated lime (%)(L)	−10.082	3.156	−3.193	<0.001	−16.321	−3.834
	Hydrated lime (%)(Q)	−0.185	0.013	−14.125	<0.001	−0.213	−0.165
	1L × 2L	3.161	0.512	6.165	<0.001	2. 141	4.176

**Table 10:** Evaluation of the effect of SAA modified foamed bitumen with and hydrated lime in asphalt concrete on ITSR.

Effect	Variable: ITSR (%); ANOVA; R^2^ = 0.597; R^2^ -adj = 0.583, MS = 8.969			
	SS	MS	F	*p*-Value
(1)Foamed bit.(%)(L)	1077.602	1077.602	120.147	<0.001
Foamed bit.(%)(Q)	15.831	15.828	1.764	0.186
(2)Hydr. Lime (%)(L)	122.404	122.399	13.647	<0.001
Hydr. Lime (%)(Q)	601.278	601.279	67.039	<0.001
1L × 2L	23.327	23.333	2.602	0.109
Error	581.411	8.969	-	-
Total SS	3078.169	-	-	-

**Table 11:** Parameters of the model of the relationship between ITSR and the amounts of foamed bitumen with 0.6% SAA and hydrated lime.

Response	Effect	Param.	SE	t(18)	*p*-Value	−95% Cnf. Lmt	+95% Cnf. Lmt
*ITSR* R^2^ = 0.597	Intercept	−77.014	101.467	−0.759	0.449	−277.641	123.625
	(1)Foamed bit. (%)(L)	51.120	33.576	1.522	0.130	−15.269	117.521
	Foamed bit. (%)(Q)	−3.681	2.773	−1.328	0.186	−9.161	1.800
	(2)Hydrated lime (%)(L)	0.032	0.273	0.112	0.910	−0.513	0.571
	Hydrated lime (%)(Q)	−0.011	0.001	−8.188	<0.001	−0.009	−0.007
	1L × 2L	0.069	0.044	1.613	0.109	−0.023	0.159

**Table 12:** Summary of the results of indirect tensile strength tests, ITS_d_^A^ and ITS_w_^A^ (modified AASHTO T283) for AC 8 S.

Variables		ITS_d_^A^ (kPa)			ITS_w_^A^ (kPa)		
Foamed Bitumen with 0.6% SAA (%)	Hydrated Lime (%)	X	s	ν (%)	X	s	ν (%)
5.6	0	940.0	26.26	2.79	747.8	21.79	2.91
	15	989.3	32.49	3.28	804.1	13.17	1.63
	30	1050.8	50.40	4.79	880.9	44.77	5.08
	45	950.5	42.62	4.48	786.3	34.77	4.42
5.9	0	917.3	44.01	4.79	747.1	37.96	5.08
	15	953.1	38.55	4.04	793.4	32.36	4.07
	30	1024.1	32.00	3.12	872.7	28.04	3.21
	45	941.6	35.83	3.80	791.1	24.14	3.05
6.2	0	873.0	38.95	4.46	717.3	31.58	4.40
	15	925.4	39.55	4.27	781.2	35.77	4.57
	30	967.8	55.74	5.76	832.1	49.00	5.88
	45	907.8	37.51	4.13	759.6	31.54	4.15
6.5	0	832.1	32.55	3.91	700.8	25.87	3.69
	15	879.9	31.65	3.59	754.9	27.11	3.59
	30	913.2	45.50	4.98	796.9	43.36	5.44
	45	878.9	40.68	4.62	755.5	32.56	4.30

**Table 13:** Evaluation of the effect of foamed bitumen with SAA and hydrated lime in asphalt concrete on ITS_d_^A^ and ITS_w_^A^.

**Effect**	**Variable: ITS_d_^A^ (kPa); ANOVA; R^2^ = 0.618, R^2^ -adj = 0.605, MS = 1576.415**			
	**SS**	**MS**	**F**	***p*-Value**
(1) Foamed bit. (%) (L)	233,795.134	233,795.134	148.308	<0.001
Foamed bit. (%) (Q)	3208.315	3208.315	2.035	0.156
(2) Hydr. Lime (%) (L)	34,926.742	34,926.742	22.155	<0.001
Hydr. Lime (%) (Q)	120,229.852	120,229.852	76.267	<0.001
1L × 2L	1353.443	1353.443	0.858	0.355
Error	40,506.911	4050.737	2.569	<0.001
Total SS	201,781.103	1576.426		
**Effect**	**Variable: ITS_w_^A^ (kPa); ANOVA; R^2^ = 0.598, Adj. R^2^ = 0.581, MS = 1108.775**			
	**SS**	**MS**	**F**	***p*-Value**
(1) Foamed bit.(%) (L)	62,796.635	62,796.635	56.636	<0.001
Foamed bit. (%) (Q)	2549.425	2549.425	2.299	0.131
(2) Hydr. Lime (%) (L)	69,696.716	69,696.716	62.859	<0.001
Hydr. Lime (%) (Q)	146,593.351	146,593.351	132.212	<0.001
1L × 2L	1.801	1.801	0.001	0.967
Error	47,294.301	4729.446	4.265	<0.001
Total SS	1,141,923.002	1108.811	-	-

**Table 14:** Parameters of the model of the relationship between ITS_d_^A^, ITS_w_^A^, and the amounts of foamed bitumen with 0.6% SAA and hydrated lime.

Response	Effect	Parameter	SE	t(18)	p-Value	−95% Cnf. Lmt	+95% Cnf. Lmt
*ITS_d_^A^* R^2^ = 0.618	Intercept	−228.459	1345.211	−0.169	0.86541	−2890.191	2433.271
	(1)Foamed bit. [%] (L)	502.201	445.139	1.128	0.26135	−378.582	1382.985
	Foamed bit. [%] (Q)	−52.446	36.763	−1.426	0.15613	−125.194	20.296
	(2)Hydrated lime [%] (L)	3.410	3.625	0.940	0.34859	−3.765	10.583
	Hydrated lime [%] (Q)	−0.128	0.015	−8.733	< 0.001	−0.161	−0.099
	1L × 2L	0.545	0.588	0.926	0.35589	−0.627	1.709
*ITS_w_^A^* R^2^ = 0.598	Intercept	−605.351	1128.175	−0.536	0.59249	−2837.641	1626.937
	(1)Foamed bit. [%] (L)	502.984	373.321	1.347	0.18025	−235.694	1241.662
	Foamed bit. [%] (Q)	−46.752	30.832	−1.516	0.13189	−107.763	14.254
	(2)Hydrated lime [%] (L)	7.572	3.040	2.490	0.01409	1.566	13.588
	Hydrated lime [%] (Q)	−0.142	0.012	−11.498	<0.001	−0.175	−0.117
	1L × 2L	0.020	0.493	0.040	0.96776	−0.961	0.996

**Table 15:** Evaluation of the effect of foamed bitumen with SAA and hydrated lime in asphalt concrete AC 8 S on RW_WM_ (ANOVA).

Effect	Variable: R (%); ANOVA; R^2^ = 0.757; R^2^ -adj : 0.748, MS = 1.021			
	SS	MS	F	*p*-Value
(1)Foamed bit.(%)(L)	278.636	278.636	230.195	<0.001
Foamed bit.(%)(Q)	0.151	0.151	0.125	0.724
(2)Hydrated Lime (%)(L)	134.503	134.503	111.120	<0.001
Hydrated Lime (%)(Q)	98.210	98.209	81.136	<0.001
1L × 2L	8.586	8.586	7.093	0.008
Error	36.298	3.629	-	-
Total SS	687.124	1.022	-	-

**Table 16:** Parameters of the model of the relationship between RW_WM_ and the amount of foamed bitumen with 0.6% SAA and hydrated lime (modified AASHTO T283).

Response	Effect	Parameter	SE	t(18)	*p*-Value	−95% Cnf. Lmt	+95% Cnf. Lmt
*RW_WM_* R^2^ = 0.76	Intercept	63.799	37.276	1.712	0.089	−9.907	137.504
	(1) Foamed bit. (%) (L)	0.775	12.335	0.063	0.950	−23.615	25.164
	Foamed bit. (%) (Q)	0.360	1.019	0.353	0.728	−1.655	2.374
	(2) Hydrated lime (%) (L)	0.485	0.100	4.833	<0.001	0.287	0.684
	Hydrated lime (%) (Q)	−0.004	0.000	−9.009	<0.001	−0.004	−0.003
	1L × 2L	−0.043	0.016	−2.663	0.009	−0.076	−0.011

**Table 17:** Correlations between AC 8 S parameters.

Variable	Correlations with Correlation Coefficients are Significant with *p* < 0.05		
	V_a_ (%)	ITSR (%)	RW_WM_ (%)
V_a_ (%)	1.000	−0.487	−0.464
	*p*= -	*p*= < 0.001	*p*= < 0.001
ITSR (%)	−0.487	1.000	0.698
	*p*= < 0.001	*p*= -	*p*= < 0.001
RW_WM_ (%)	−0.464	0.698	1.000
	*p*= < 0.001	*p*= < 0.001	*p*= -

**Table 18:** Parameters of the model describing the properties of AC 8 S with respect to the contents of 50/70-grade foamed bitumen and hydrated lime.

Dependent Variable	SS Test for the Full Model with Respect to SS for Residuals								
	Multicrit. R	Multicrit. R^2^	Adjusted. R^2^	SS Model	MS Model	SS Residual	MS Residual	F	*p*
V_a_ (%)	0.908	0.826	0.821	1.043 × 10^2^	26	22	0	165.1	<0.001
ITSR (%)	0.768	0.590	0.578	1.817 × 10^3^	454	1261	9	50.0	<0.001
RW_WM_ (%)	0.62	0.744	0.737	5.115 × 10^2^	128	176	1	101.2	<0.001

## References

[1] Vaitkus A., Čygas D., Laurinavičius A., Perveneckas Z. (2009). Analysis and evaluation of possibilities for the use of warm-mix asphalt in Lithuania. Balt. J. Road Bridge Eng..

[2] Ozturk H.I., Kutay M.E. (2014). Novel Testing Procedure for Assessment of Quality of Foamed Warm Mix Asphalt Binders. J. Mat. Civ. Eng..

[3] Leng Z., Gamez A., Al-Qadi I.L. (2014). Mechanical property characterization of warm-mix asphalt prepared with chemical additives. J. Mater. Civ. Eng..

[4] Król J., Kowalski K., PRadziszewski P. (2015). Rheological behavior of n-alkane modified bitumen in aspect of Warm Mix Asphalt technology. Constr. Build. Mater..

[5] Sanchez-Alonso E., Vega-Zamanillo A., Castro-Fresno D., Del Rio-Prat M. (2011). Evaluation of compatibility and mechanical properties of bituminous mixes with warm additives. Constr. Build. Mater..

[6] Pszczoła M., Jaczewski M., Rys D., Jaskula P., Szydlowski C. (2018). Evaluation of Asphalt Mixture Low-Temperature Performance in Bending Beam Creep Test. Materials.

[7] Remišová E., Holý M. (2017). Changes of Properties of Bitumen Binders by Additives Application 2017 IOP Conf. Ser. Mater. Sci. Eng..

[8] Silva H.M., Oliveira J.R., Peralta J., Zoorob S.E. (2010). Optimization of warm-mix asphalt using different blends of binders and synthetic paraffin wax contents. Constr. Build. Mater..

[9] Jamshidi A., Hamzah M.O., You Z. (2013). Performance of Warm Mix Asphalt containing Sasobit®: State-of-the-art. Constr. Build. Mater..

[10] Iwański M., Mazurek G. The influence of the low-viscosity modifier on viscoelasticity behavior of the bitumen at high operational temperature. Proceedings of the 8th International Conference Environmental Engineering.

[11] Iwański M., Mazurek G. (2015). Effect of Fischer-Tropsch synthetic wax additive on the functional properties of bitumen. Polimery.

[12] Lu X., Redelius P. (2006). Effect of bitumen wax on asphalt performance. Constr. Build. Mater..

[13] Sengoz B., Topa A., Gorkem C. (2013). Evaluation of natural zeolite as warm mix asphalt additive and its comparison with other warm mix additives. Const. Build. Mater..

[14] Woszuk A., Wojciech Franus W. (2017). A Review of the Application of Zeolite Materials in Warm Mix Asphalt Technologies. Appl. Sci..

[15] Woszuk A., Zofka A., Bandura L., Franus W. (2017). Effect of zeolite properties on asphalt foaming. Const. Build. Mater..

[16] Woszuk A., Franus W. (2016). Properties of the Warn Mix Asphalt involving clinoptilolite and Na-P1 zeolite additives. Constr. Build. Mater..

[17] Barthel W., Marchand J., Von Devivere M. (2004). Warm Mix Asphalt by adding a synthetic zeolite. Proceedings of the Third Eurasphalt and Eurobitume Conference.

[18] Van De Ven M.F.C., Jenkins K.U., Voskuilen J.L.M., Van Den Beemt R. (2007). Development of (half-) warm foamed bitumen mixes: State of the art. Int. J. Pavement Eng..

[19] Jenkins K.J., de Groot J.L.A., Van de Ven M.F.C., Molenaar A. Half-warm Foamed Bitumen Treatment, A New Process. Proceedings of the Conference on Asphalt pavements for Southern Africa.

[20] Jenkins K.J. (2000). Mix Design Considerations for Cold and Half-Warm Bituminous Mixes with Emphasis on Foamed Bitumen. Ph.D. Dissertation.

[21] Muthen K.M. (2009). Foamed asphalt mixes. Mix design procedure.

[22] Yu X., Wang F., Luo T. (2013). Impacts of water content on rheological properties and performance-related behaviors of foamed war-mix asphalt. Constr. Build. Mater..

[23] You L., You Z., Yang X., Ge D., Lv S. (2018). Laboratory testing of rheological behavior of water-foamed bitumen. J. Mater. Civ. Eng..

[24] Chomicz-Kowalska A., Gardziejczyk W., Iwański M.M. Analysis of IT-CY stiffness modulus of foamed bitumen asphalt concrete compacted at 95 °C. Proceedings of the 12th International Scientific Conference of Modern Building Materials, Structures and Techniques (MBMST).

[25] Mrugała J. (2015). Optymalizacja składu betonu asfaltowego w technologii na półciepło z asfaltem spienionym w aspekcie właściwości eksploatacyjnych. [Optimization of asphalt concrete composition in half-warm mix asphalt technology with foamed bitumen in the aspect of operational properties]. Ph.D. Thesis.

[26] You L., You Z., Dai Q., Guo S., Wang J., Schiltz M. (2018). Characteristics of water-foamed asphalt mixture under multiple freeze-thaw cycles: Laboratory evaluation. J. Mater. Civ. Eng..

[27] Iwański M., Chomicz-Kowalska A. Evaluation of the effect of using foamed bitumen and bitumen emulsion in cold recycling technology. Proceedings of the 3rd International Conference on Transportation Infrastructure (ICTI) Sustainability, Eco-efficiency and conservation in transportation infrastructure asset management.

[28] Buczyński P., Iwański M. (2017). Inactive Mineral Filler as a Stiffness Modulus Regulator in Foamed Bitumen-Modified Recycled Base Layers. IOP Conf. Series Mater. Sci. Eng..

[29] Wirtgen (2012). Wirtgen Cold Recycling Technology.

[30] Iwański M., Chomicz-Kowalska A., Maciejewski K. (2015). Application of synthetic wax for improvement of foamed bitumen parameters. Constr. Build. Mater..

[31] Mrugała J., Iwański M.M. (2015). Resistance to permanent deformation of asphalt concrete with F-T wax modified foamed bitumen. Proceedings of the 7th Scientific-Technical Conference Material Problems in Civil Engineering (MATBUD2015).

[32] Iwański M.M., Chomicz-Kowalska A., Maciejewski K. (2019). Effect of Surface Active Agent (SAA) on 50/70 Bitumen Foaming Characteristics. Materials.

[33] Stefańczyk B., Mieczkowski P. (2008). Mieszanki mineralno-asfaltowe: Wykonawstwo i badania. Bituminous Mixtures: Performance and Research.

[34] Seebaly P.E., Litte D.N., Epps J.A. (2006). The Benefis of Hydrated Lime in Hot Mix Asphalt.

[35] Chachas C.V., Liddle W.J., Petersom D.E., Wiley M.L. (1971). Use of Hydrated Lime in Bituminous Mixtures to decrease Hardening of the Asphalt Cement.

[36] Petresen J.C., Plancher H., Hansberg P.M. Lime treatment of asphalt to reduce age hardening and improve flow properties. Proceedings of the Association Asphalt Paving Technologists.

[37] Stroup-Gardiner M., Epps J.A. Effect of Lime on Asphalt Concrete Performance. Proceedings of the American Society Civil Engineers Materials Congress.

[38] Luxemburg F. Lime Hydrate as an Additive to Improve the Adhesion of Bitumen to the Aggregates. Proceedings of the II International Conference Durable and Save Road Pavements.

[39] Lesueur D., Petit J., Ritter H.-J. (2012). The mechanism of hydrated lime modification of asphalt mixtures: A state-of-the-art review. Road Mater. Pavement Des..

[40] Iwański M. (2014). Wapno hydratyzowane wielofunkcyjnym dodatkiem zwiększającym trwałość nawierzchni SMA. (Hydrated lime as a multifunctional additive for improving the durability of SMA pavements). Politechnika Świętokrzyska.

[41] Plancher H., Green E.L., Petersen J.C. Reduction of oxidation hardening of asphalts by treatment with hydrated lime – A mechanistic study. Proceedings of the Association Asphalt Paving Technologists 45.

[42] Gorkem C., Sengoz B. (2008). Predicting stropping and moisture induced damage of asphalt concrete prepared with polymer modified bitumen and hydrated lime. Constr. Build. Mater..

[43] Lesueur D., Little D.N. (1999). Effect on hydrated lime on rheology, fracture and aging of bitumen. Transp. Res. Rec..

[44] Hanson D.I., Graves R.E., Brown E.E. (1994). Laboratory evaluation of the addition of lime treated sand to hot-mix asphalt. Transp. Res. Rec..

[45] Das A.K., Singh S.D. (2017). Investigation of rutting, facture and thermal cracking behavior of asphalt mastic containing basalt and hydrated lime fillers. Constr. Build. Mater..

[46] Zou J., Isola M., Roque R., Chun S., Koh C., Lopp G. (2013). Effect of hydrated lime on fracture performance of asphalt mixture. Constr. Build. Mater..

[47] Iwański M., Mazurek G. (2016). Rheological properties of the bituminous binder extracted from SMA pavement with hydrated lime. Balt. J. Road Bridge Eng..

[48] Iwański M., Mazurek G. Hydrated lime as the anti-aging bitumen agent. Proceedings of the 11th International Scientific Conference of Modern Building Materials, Structures and Techniques (MBMST).

[49] Das A.K., Singh D. (2018). Effects of Basalt and Hydrated Lime Fillers on Rheological and Fracture Cracking Behavior of Polymer Modified Asphalt Mastic. J. Mater. Civ. Eng..

[50] Jaskula P., Judycki J. (2008). Verification of the criteria for evaluation of water and frost resistance of asphalt concrete. Road Mater. Pavement Des..

[51] Judycki J., Jaskuła P. (1997). Badania odporności betonu asfaltowego na oddziaływanie wody i mrozu. (Investigations on the moisture and frost resistance of asphalt concrete). Drogownictwo.

[52] Hesami S., Roshani H., Ossein Hamedi G., Azarhoosh A. (2013). Evaluate the mechanism of effect of hydrate lime on moisture damage of warm mix asphalt. Constr. Build. Mater..

[53] Khadaii A., Kaziem Tehrani H., Haghshenas H.F. (2012). Hydrated lime effect on moisture susceptibility of warm mix asphalt. Constr. Build. Mater..

[54] Remišová E., Decký M., Podolka L., Kováč M., Vondráčková T., Bartuška L. (2015). Frost Index from Aspect of Design of Pavement Construction in Slovakia. Procedia Earth Planet. Sci..

[55] Jahromi S.G. (2009). Estimation of resistance to moisture destruction in asphalt mixtures. Constr. Build. Mater..

[56] Trojanová M., Decký M., Remišová E. (2015). The Implication of Climatic Changes to Asphalt Pavement Design. Proceedings of the XXIV R-S-P seminar, Theoretical Foundation of Civil Engineering (24RSP) (TF°CE 2015).

[57] Judycki J., Jaskuła P., Pszczoła M., Alenowicz J., Dołżycki B., Jaczewski M., Ryś D., Stienss M. (2014). Katalog typowych konstrukcji nawierzchni podatnych i półsztywnych (Catalogue of Typical Flexible and Semi-Rigid Pavement Constructions).

[58] WT-2 (2014). Technical Guidelines 2: Asphalt pavements for national roads. Part I: Asphalt mixes.

[59] Pralińska M., Praliński J. (2007). Badania statystyczne z Excelem. (Investigations in Statistics with Excel).

[60] Koronacki J., Mielniczuk J. (2004). Statystyka dla Studentów kierunków Technicznych i przyrodniczych. (Statistics for Technical and Natural Sciences Students).

[61] Iwański M., Uriew N.B. (2007). The Asphalt Concrete as A Composite Material (with Nanodisperse and Polimer Components).

[62] Piasta Z., Lenarcik A. (1998). Methods of Statistical Multi-Criteria Optimization.

[63] STATISTICA 13.3 Statsoft. www.statsoft.com.

[64] (2016). National Centre for Research and Development (NCBR) and the Polish General Directorate for National Roads and Motorways (GDDKIA).

[65] Chomicz-Kowalska A., Maciejewski K. (2015). Multivariate optimization of recycled road base cold mixtures with foamed bitumen. Procedia Eng..

